# The Fate of the Nucleolus during Mitosis: Comparative Analysis of Localization of Some Forms of Pre-rRNA by Fluorescent in Situ Hybridization in NIH/3T3 Mouse Fibroblasts

**Published:** 2011

**Authors:** K.V. Shishova, О.О. Zharskaya, О.V. Zatsepina

**Affiliations:** Shemyakin and Ovchinnikov Institute of Bioorganic Chemistry, Russian Academy of Sciences

**Keywords:** nucleolus, mitosis, nucleolus-derived foci (NDF), NIH/3Т3 mouse fibroblasts, fluorescence*in situ*hybridization (FISH)

## Abstract

Nucleolus is the major structural domain of the cell nucleus, which in addition to proteins contains ribosomal RNA (rRNA) at different stages of maturation (or pre-rRNA). In mammals, the onset of mitosis is accompanied by the inhibition of rRNA synthesis, nucleolus disassembly, and the migration of pre-rRNA to the cytoplasm. However, the precise role of cytoplasmic pre-rRNA in mitosis remains unclear, and no comparative analysis of its different forms at consequent mitotic stages has thus far been performed. The focus of this research was the study of the localization of pre-rRNA in mitotic NIH/3T3 mouse fibroblasts by fluorescent*in situ*hybridization (FISH) with probes to several regions of mouse primary 47S pre-rRNA transcripts and by confocal laser microscopy. The results reveal that all types of pre-rRNA appear in the cytoplasm at the beginning of mitosis, following the breakdown of the nucleolus and nuclear envelope. However, not all pre-rRNA are transported by chromosomes from maternal cells into daughter cells. At the end of mitosis, all types of pre-rRNA and 28S rRNA can be visualized in nucleolus-derived foci (NDF), structures containing many proteins of mature nucleoli the appearance of which indicates the commencement of nucleologenesis. However, early NDF are enriched in less processed pre-RNA, whereas late NDF contain predominantly 28S rRNA. Altogether, the results of this study strengthen the hypotheses that postulate that different forms of pre-rRNA may play various roles in mitosis, and that NDF can be involved in the maturation of pre-rRNA, remaining preserved in the cytoplasm of dividing cells.

## INTRODUCTION

**Fig. 1:**
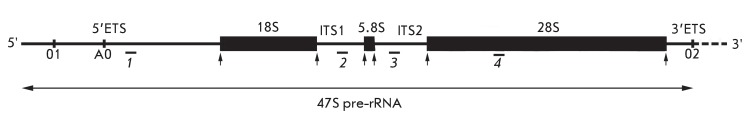
Structure of primary mouse transcript (47S pre-rRNA) and location of probes for *in situ* hybridization. 5’ETS – 5’-external transcribed spacer; ITS1 – internal transcribed spacer 1; ITS2 – internal transcribed spacer 2; 3’-ETS – 3’-external transcribed spacer. 18S, 5.8S, 28S – coding regions of pre-rRNA. The sequence location of probes for *in situ* hybridization is shown below the 47S pre-rRNA diagram: for 5’ETS +2251/+2280 (probe  *1* ); for ITS1 +6391/+6420 (probe *2* ); for ITS2 – +7471/7500 (probe *3* ), for 28S rRNA – +9571/9600 (probe *4* ). 01, 02, A0 – endonucleolytic cleavage sites in pre-rRNA.

Nucleolus is the major structural domain of the cell nucleus, whereby the transcription of ribosomal genes (rDNA), the processing (maturation) of primary transcripts (pre-RNA), and the assembly of the ribosomal particles occur [1, 2]. In mammalian cells, three types of cytoplasmic rRNA (18S, 5.8S, and 28S) are synthesized in nucleoli in the form of the common precursor 46S pre-rRNA. Maturation of 47S pre-rRNA into rRNA is a complex multistage process which includes the excision of several spacer fragments transcribed within 47S pre-RNA (5’-external transcribed spacer (5’ETS), as well as the first (ITS1) and the second (ITS2) internal transcribed spacers) in addition to the chemical modifications of 18S, 5.8S, and 28S rRNAs ( *Fig* . 1). It is known that the maturation time of 18S rRNA and 28S rRNA is 20 and 40 min, respectively. Consequently, in addition to primary rRNA transcripts, partially processed pre-rRNA of varying size are also found in the fraction of isolated nucleoli [3]. In mice, a 650 bp fragment located at the 5’-terminus of ETS is the shortest lived one, its half-life being less than 2 min [4]. According to the existing notion, the excision of internal spacer in mammals begins following completion of the synthesis and detachment of the primary pre-rRNA transcript from the matrix rRNA. The half-life of the internal spacers in mammals is at least 30 min [3–5]. 

It is well known that mitosis in higher eukaryotes is accompanied by the termination of pre-rRNA synthesis, the disassembly of the nucleolus, and the migration of the major nucleolar components, proteins and rRNA, into the cytoplasm [6–10]. 

The methods of biochemical [11, 12] and cytological analysis [13] were used to demonstrate that pre-rRNA synthesized before mitosis remains preserved in the cellular cytoplasm up to its completion. However, the role of this stable pre-rRNA in mitosis has yet to be elucidated. The features of localization of different pre-rRNA forms in mitosis have not been sufficiently studied, although research in this area will shed light on their role in the recovery of nucleoli during the latter stages of mitosis.

The recovery of nucleoli during mitotic cell division begins immediately after the chromosomes separate and move to the mitotic spindle poles and numerous discrete bodies (of 0.2–2.0 µm diameter), and the so-called nucleolus-derived foci (NDF) emerge in the cytoplasm. Currently, NDF are reported to include numerous proteins of mature nucleoli participating in pre-rRNA processing (B23/nucleophosmin, C23/nucleolin, fibrillarin, etc.), U3 and U14 small nucleolar RNA (snoRNA), as well as mature 18S and 28S rRNA). The methods of immunocytochemistry [13, 14] and the expression of protein markers of NDF fused with fluorescent proteins were used in order to demonstrate the gradual decrease in the amount of NDF-containing proteins of early pre-rRNA processing (e.g., fibrillarin) following the completion of mitosis. On the contrary, the proteins participating in the late stages of pre-rRNA processing (e.g., B23/nucleophosmin) are retained among NDF up to the G1 phase of the subsequent cell cycle [15]. The presence of the proteins and snoRNAs, which are required for pre-rRNA processing in interphase nucleoli, among NDF allows one to reasonably assume that at least some of the stages of maturation of pre-rRNA (which remains preserved in cells during mitosis) can take place in NDF. However, at the time of writing no experiments have been performed to verify this assumption. The presence of different forms of pre-rRNA in early and late NDF has been insufficiently studied.

The major aim of this study was to perform a comparative analysis of the localization of different forms (intermediates) of partially processed pre-rRNA and 28S rRNA at sequential phases of mitosis in NIH/3T3 mouse fibroblasts via fluorescent *in situ * hybridization and confocal laser microscopy. 

## EXPERIMENTAL

**Cell culture**

 NIH/3T3 mouse fibroblasts were obtained from the Russian Cell Culture Collection of the Institute of Cytology of the Russian Academy of Sciences; the cells were free of micoplasma. The cells were cultured in a DMEM medium (PanEco, Russia) containing 10% fetal bovine serum (HyClone, USA), 2 mM  *L* -glutamine, penicillin and streptomycin (250 U of each antibiotic) at 37 ^0^ С and 5% CO _2_ , with re-culturing twice a week. 

**Fluorescent in situ hybridization**

In this study, we used oligonucleotide probes labelled by biotin at the 5’-terminus, which were capable of specific detection of the following fragments of mouse 47S pre-rRNA: the core fragment of the 5’-external transcribed spacer (5’ETS, probe  *1* ) – 5’aga gag aga ccg atg ccg aca cac cga tgc (+2251/+2280); the first internal transcribed spacer (ITS1, probe  *2* ) – 5’aaa cct ccg cgc cgg aac gcg aca gct agg (+6391/+6420); the second internal transcribed spacer (ITS2, probe *3* ) – 5’cag aca acc gca ggc gac cga ccg gcc (+7471/+7500); and a 28S rRNA fragment (probe  *4* ) 5’gag gga acc agc tac tag atg gtt cga tta (+9571/+9600). The probes were synthesized by Sintol (Russia); the probe concentration in stock solutions was approximately equal to 2 µg/µl. The localization of probes with respect to the mouse 47S pre-rRNA is shown in *Fig. 1* . As can be seen in *Fig.*   *1, * probe  *1 * detected the less processed pre-rRNA form; probes *2* and *3* could hybridize both with longer or shorter (i.e., processed to a larger extent) forms of pre-rRNA; probe  *4* mainly detected the mature 28S rRNA, but it could also hybridize with the immature pre-rRNA as well. 

**Fig. 2:**
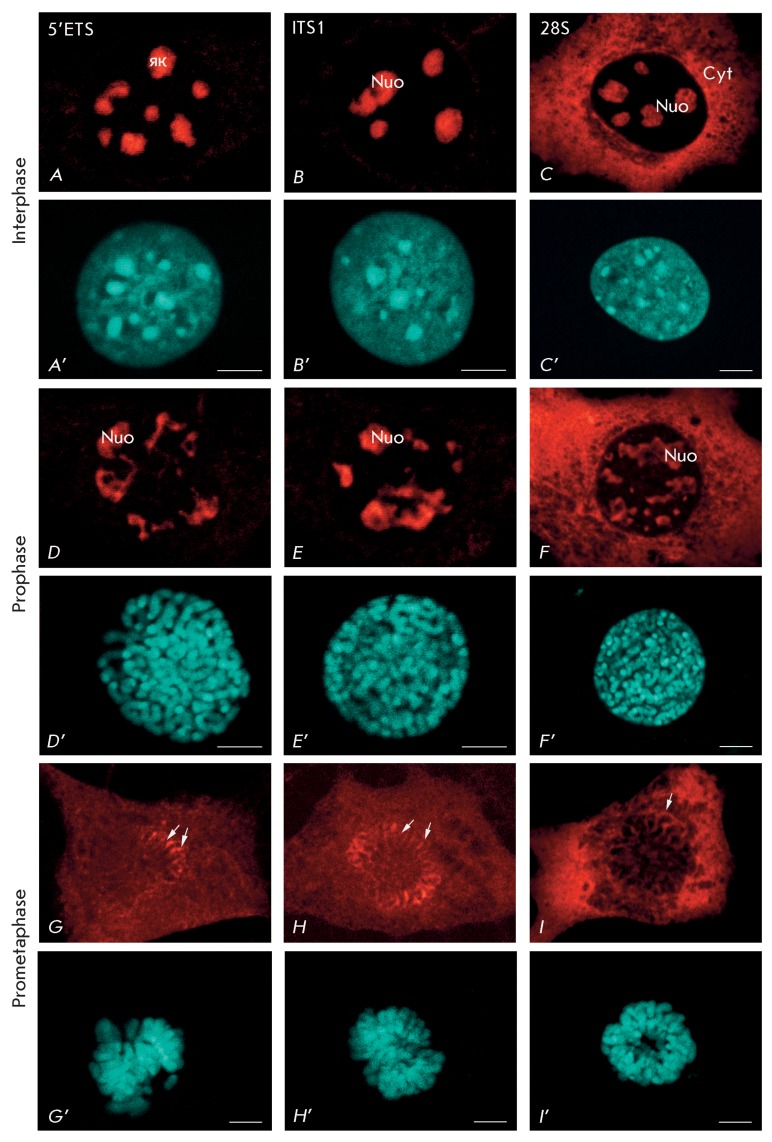
Pre-rRNA location in NIH/3T3 cells detected by fluorescent *in situ* hybridization with probes to 5’ETS (probe  *1* ) ( *A* , *D* , *G* ), ITS1 (probe  *2* ) ( *B* , *E* , *H* ), and 28S rRNA (probe  *4* ) ( *C* , *F* , *I* ) in interphase ( *A* – *C* ), prophase ( *D* – *F* ) and prometaphase ( *G* – *I* ). ( *A* – *I* ) – pre-rRNA and 28S location; ( *A* ’– *I* ’) – chromatin staining with DAPI in interphase and chromosomes in mitosis. nuo – nucleoli; cyt – cytoplasm; arrows – perichromosomal material. Bars, 5 µm.

The cells grown on coverslips were washed with a phosphate saline buffer (PSB, 140 mM NaCl, 2.7 mM KCl, 1.5 mM KH _2_ PO _4_ , and 8.1 mM Na _2_ HPO _4_ , рН 7.2–7.4), followed by subsequent fixation with a 4% formalin solution (MP Biomedicals, Inc., France) in PBS for 30 min at room temperature. The cells were then washed with PBS (3 × 5 min), treated with 0.5% Triton X-100 (10 min at 4 ^о^ С), washed with PBS, followed by a two-fold washing with a standard saline buffer (2×SSC, 0.3 M NaCl, 0.03 M Na _3_ С _6_ Н _5_ О _7_ , рН 7.0) for 5 min. 

The hybridization mixture was composed of 50% of deionized formamide (Sigma, USA), 10% of dextran sulphate (Loba Chemie, Fischamend, Austria), 5% of 20×SSC (3 M NaCl, 0.3 M Na _3_ С _6_ Н _5_ О _7_ , рН 7.0), and 8 ng/µl oligo samples. Hybridization was performed in a wet chamber for 16 h at 42 ^о ^ C. The cells were then sequentially washed with 50% formamide (Panreac, Spain) in 2×SSC (3 × 10 min) at 42 ^о^ C, 2×SSC at 42 ^о^ C (10 min), and 2×SSC (1 min) at room temperature. The hybridization sites were detected using rhodamine-conjugated avidin (Roche, Switzerland) after 1 : 200 dilution in the buffer containing 4×SSC (0.06 M NaCl, 0.06 M Na _3_ С _6_ Н _5_ О _7_ , рН 7.0) for 1 h at room temperature. The cells were then washed with 4×SSC (10 min) and PBS (3 × 10 min). Chromatin and chromosomes were stained with a DAPI dye (1 µg/ml, 4’-6-diamidino-2’-phenylindole, Sigma) for 10 min. The cells were embedded in Mowiol (Calbiochem, USA) and examined on an LSM510 DuoScanMETA confocal laser scanning microscope (Carl Zeiss, Germany) equipped with argon (Ar) and helium-neon (He-Ne) lasers, using a Plan-Apochromat 63×/1.40 numerical aperture immersion lens. In order to obtain the control sample, the fixed cells were treated with RNAse A (200 µg/ml) in PBS for 30 min at 37 ^о^ C according to the previously described procedure [16]. The treatment with RNAse A resulted in the complete blockage of the emergence of fluorescent signals in nucleoli during interphase and of the mitotic signals after FISH was performed (not shown). A minimum of 20 cells for the control and experimental samples were analyzed for each stage. 

## RESULTS AND DISCUSSION

**Fig. 3:**
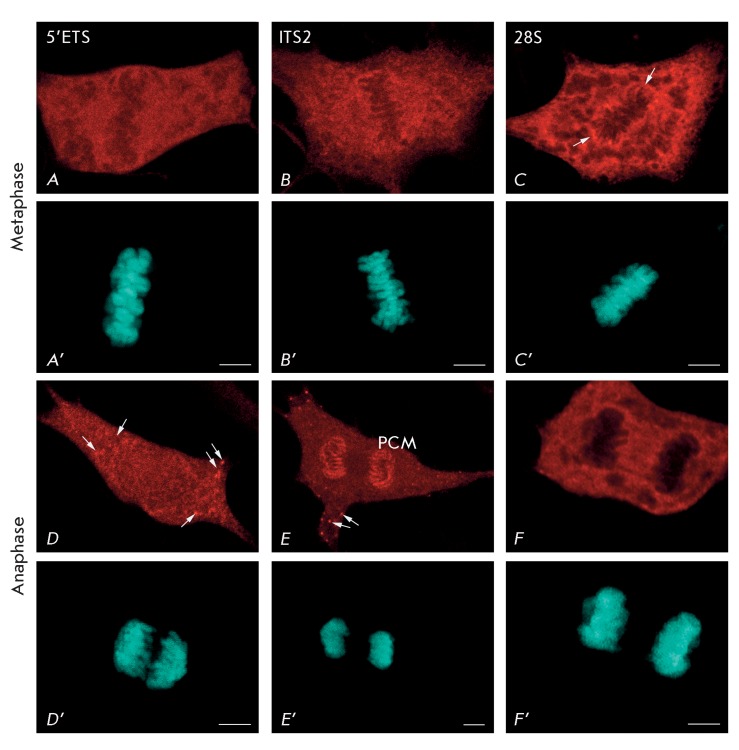
Pre-rRNA location in NIH/3T3 cells detected by FISH with probes to 5’ETS (probe  *1* ) ( *A* , *D* , *G* ), ITS2 (probe *3* ) ( *B* , *E* , *H* ), and 28S rRNA (probe  *4* ) ( *C* , *F* , *I* ) in metaphase ( *A* – *C* ) and anaphase ( *D* – *F* ). ( *A* – *F* ) – pre-rRNA and 28S location; ( *A* ’– *F* ’) – chromatin and chromosome staining with DAPI. PCM – peripheral chromosomal material; arrows ( *D* , *E* ) – nucleoli-derived foci (NDF); arrows ( *C* ) – PCM. Bars, 5 µm.

The localization of pre-rRNA and 28S rRNA in interphase cells NIH/3T3 is shown in *Fig. 3* . It is clear that all pre-rRNA forms were detected in nucleoli only ( *Fig. 2A,B* ), whereas 28S rRNA was detected both in the nucleolus and the cytoplasm of mature ribosomes ( *Fig. 2C* ). These observations are in close agreement with data published by other authors [5, 7, 14]; however, the hybridization signals in this case were brighter and more distinct. We believe that this can be accounted for both by the efficiency of the labelling of oligonucleotide probes and by the conditions of the FISH experiment, including the parameters of the washing in the buffer, which enabled the removal of the unbound probes, thereby reducing the background (nonspecific) fluorescence. 

**Fig. 4:**
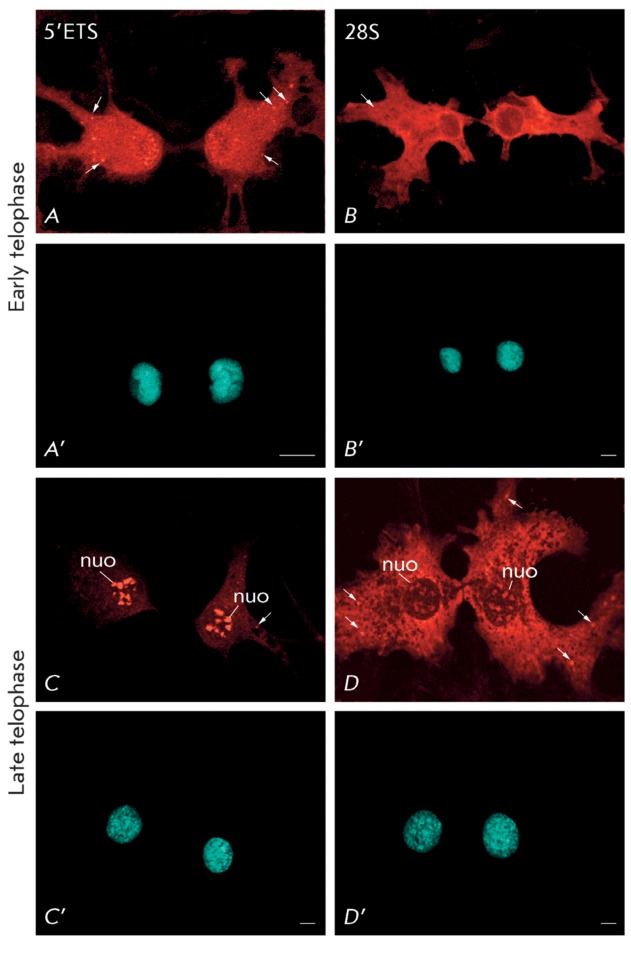
Pre-rRNA location in NIH/3T3 cells detected by FISH with probes to 5’ETS (probe  *1* ) ( *A* , *C* ) and 28S rRNA (probe  *4* ) ( *B* , *D* ) in early telophase ( *A* , *B* ) and late telophase ( *C* , *D* ). ( *A* – *D* ) – pre-rRNA and 28S location; ( *A* ’– *D* ’) – chromatin and chromosome staining with DAPI. nuo – nucleoli; arrows – nucleoli-derived foci (NDF). Bars, 10 µm.

At the initial mitotic stage (during prophase), the cells were identified based on the presence of long condensed chromosomes, which were distinctly detected by the DAPI dye in nuclei ( *Fig. 2G’–E’* ). It was known that all the proteins participating in pre-rRNA processing migrate from the nucleoli into the nucleus during prophase, and that they are diffusely arranged between chromosomes [9, 16–18]. Among these proteins are the following: fibrillarin (the early pre-rRNA processing factor) [19], B23/nucleophosmin (the ribosome assembly factor) [20], and SURF-6 (the late pre-rRNA processing factor) [16, 17]. The results obtained in this study show that the immature rRNAs detected by probes to 5’ETS ( *Fig. 2D* ), ITS1 ( *Fig. 2E* ), ITS2 (not shown), and 28S rRNA ( *Fig. 2F* ) were mostly located in the nucleolar area and were quasi absent in the nucleus during prophase, as opposed to proteins. No differences were detected in the localization of the pre-rRNAs revealed by probes to ITS1 and ITS2. The differences in the behavior of the pre-rRNAs and proteins participating in its maturation during nucleolar disassembly have yet to be described. It is reasonable to assume that these differences indicate the partial disassembly of pre-rRNA–protein complexes, which accompanies the termination of the processing of the pre-rRNA that was synthesized prior to mitosis, or in the very beginning of this process. 

Nuclear envelope disassembly relates to the progression of cells from prophase into metaphase. It is marked by additional condensation of chromosomes and the alteration of the contour of the area occupied by them. It is known that nucleolar disassembly is terminated and most nucleolar proteins migrate to the cytoplasm during prometaphase [15]. According to the results obtained in this study, all pre-rRNA forms can be distinctly detected in the cytoplasm and on the chromosome surface during prometaphase ( *Fig. 2G,H* ). However, the fluorescent signals detected by a probe to 5’ETS were present on the surface of only a number of chromosomes ( *Fig. 2G* ), whereas the signals detected by the probes to ITS1 ( *Fig. 2H* ) and ITS2 (not shown) could be seen on the surface of all chromosomes. An identical pattern was also observed during the subsequent stage of mitosis (metaphase), when the chromosomes formed a characteristic plate at the center of the cell ( *Fig. 3A–B’* ). However, the best defined distinctions in the localization of different pre-rRNA forms can be observed during anaphase, when chromosomes separate and move to the spindle poles ( *Fig. 3D–F’* ). It is clear from the comparison of *Figs. 3D* and *3E* that the probe to ITS2 brightly stains the chromosome surface, whereas almost no hybridization occurs between the probe to 5’-ETS and the chromosome surface. These observations enable one to conclude that the less mature pre-rRNA detected by the probe to 5’-ETS was not transported by chromosomes from maternal cells into daughter cells, as opposed to the more mature (short) pre-rRNA forms that were detected by the probes to ITS1 and ITS2. 

Nucleolar disassembly during prophase causes the migration of 28S rRNA, along with the processed pre-rRNA forms, into the cytoplasm ( *Fig. 2F* ). Therefore, starting with the early prometaphase, the FISH method does not allow one to distinguish between 28S rRNA of nucleolar and cytoplasmic origin. During the late prometaphase ( *Fig. 2I* ), metaphase ( *Fig. 3C* ), and anaphase ( *Fig. 3F* ), the FISH signals detected by the probe to 28S rRNA were visualized in the cytoplasm. Moreover, more intense signals in many cells could be seen on the chromosome surface in the form of perichromosomal material ( *Fig. 3C, F* ). The presence of perichromosomal material detected by the probe to 28S rRNA can be accounted for by the presence of either mature pre-rRNA or immature 28S rRNA. This assumption agrees with the data of an *in situ* analysis of mitotic chromosomes using electron microscopy. According to these data, RNP particles of a size corresponding to that of ribosomes are located on the chromosome surface. These particles are one of the major structural components of the so-called perichromosomal material (or perichromosomal layer) [21]. It has been known that the nucleolar proteins that constitute the perichromosomal material are used to promote the formation of the nucleoli of daughter cells. On the contrary, protein material, not being a component of the perichromosomal layer, is an unlikely participant in this process [22–24]. One can assume that a similar pattern exists for different forms of pre-rRNA; i.e., less processed pre-rRNA forms (such as those detected by the probe to 5’-ETS) do not participate in nucleologenesis. 

According to current theories, one of the earliest stages in nucleolar recovery during mitosis in mammals corresponds to the formation of NDF, cytoplasmic bodies, with the proteins participating in rRNA processing as its major component [18]. However, several rRNA forms, including those of mature rRNA and pre-rRNA, have been reportedly detected within NDF, both in animal and plant cells [7, 13, 15]. The results obtained in this study unequivocally attest to the fact that mouse NDF also contain pre-rRNAs, although the labelling of NDF with the probes to various pre-rRNA forms differs for the various stages of mitosis. Early NDF (i.e., NDF during anaphase ( *Fig. 3D’–F’* ) and at the beginning of telophase ( *Fig. 4A’* )) are mostly labelled by the probe to 5’ETS ( *Figs. 3D,E; 4A* ), although they can hardly be labelled by the probe to 28S rRNA ( *Figs. 3E; 4B* ). On the contrary, during the late telophase and G1 period ( *Fig. 4C’, D’* ), NDF are detected by the probe to 28S rRNA ( *Fig. 4G* ) but cannot be labelled by the probes to 5’-ETS ( *Fig. 4C* ), ITS1, and ITS2 (not shown). It is noteworthy that the late NDF detected by the probe to 28S rRNA ( *Fig. 4D* ) are larger than those that can be detected at the same stage of mitosis by the probe to 5’-ETS ( *Fig. 4C* ). 

Based on these observations, a conclusion can be made that the composition of NDF is gradually altered during the latter stages of mitosis: the less processed pre-rRNAs disappear, while the more mature rRNAs remain preserved or are even accumulated during these stages. These observations imply the participation of NDF in the processing of pre-rRNA, which remains preserved during mitosis. It should be noted that NDF contain no rDNA; they are therefore incapable of synthesizing 47S pre-rRNA [15]. NDF are structures with a shorter lifespan in comparison with nucleoli. Therefore, if pre-rRNA processing indeed occurs within them, it occurs during a limited time period, coinciding with the termination of mitosis. The biological meaning of this phenomenon may be associated with the rational use of pre-rRNA synthesized prior to mitosis and in providing the cell with additional ribosomes at the active growth phase after mitosis.

Prenucleolar bodies (PNB) also participate in nucleologenesis at the latter stages of mitosis [15]. Similar to NDF, prenucleolar bodies are discrete formations up to 1 µm in size, which contain nucleolar rRNA processing factors. Unlike NDF, these bodies are generated not in the cytoplasm but in the daughter cell nuclei [25, 26]. Proteins are the major markers of these bodies, and the question of the presence of different pre-rRNA forms and mature rRNA in prenucleolar bodies remains poorly studied. Nevertheless, it has been shown that prenucleolar bodies in HeLa and CMT3 (green monkey) cells, as well as those in plant cells ( *Pisum sativum* and *Allium сepa* ), may contain 32S pre-rRNA and mature 28S rRNA, although the presence of 18S rRNA in prenucleolar bodies is not obvious (Table and References in [15]). Our observations is evidence that in NIH/3T3 cells prenucleolar bodies are hybridized with the same probes that hybridize with NDF, although the early PNB are hard to detect with the probe to 28S rRNA ( *Fig. 4B, B’* ). In other words, they are devoid of this form of rRNA. However, the rRNA compositions in PNBs at different stages of their existence require a special investigation. This issue could only be resolved at the cytological level, when approaches that enable one to combine high-sensitivity *in situ* hybridization with the probes to various pre-rRNA sequences, and the detection of the marker proteins of prenucleolar bodies, have been developed. 

## CONCLUSIONS

A procedure for high-sensitivity detection of different forms of pre-rRNA and mature 28S rRNA in mitotic NIH/3T3 mouse fibroblasts using biotin-labelled oligonucleotide probes was proposed. It was shown that pre-rRNA is preserved in disassembling nucleoli for a longer period of time than the proteins participating in pre-rRNA processing and that it does not disintegrate during mitosis. Only a portion of the forms of pre-rRNA were transported by chromosomes from maternal cells into daughter cells. Pre-rRNA and 28S rRNA were detected within nucleolar cytoplasmic derivatives (NDF) immediately after their formation during anaphase or early telophase. However, the disappearance of immature pre-rRNA from the NDF fabric occurred at an earlier stage than that of 28S rRNA. This observation argues for the fact that NDF participates in the processing of pre-rRNA, which is preserved in the cell cytoplasm during mitosis. 

## Abbreviations

rRNA: ribosomal RNA
47S pre-rRNA - 47S precursor of rRNA; NDF: nucleolus-derived foci
PNB: prenucleolar bodies
5’ETS, 3’ETS: 5’- and 3’-external transcribed spacers
ITS1, ITS2 - internal transcribed spacer 1 and 2; bp: base pairs
snoRNA: small nucleolar RNA
DAPI: 4’,6-diamidino-2-phenylindole
FISH: fluorescent*in situ*hybridization
